# Becoming the 'good doctor': medical students’ views on altruism and professional identity

**DOI:** 10.15694/mep.2018.0000052.1

**Published:** 2018-03-06

**Authors:** Kaat Marynissen, Bethan Spurrier

**Affiliations:** 1 University of Leeds

**Keywords:** medical education, professionalism, altruism, professional identity

## Abstract

This article was migrated. The article was marked as recommended.

Background and Purpose

Professionalism is central to modern medical practice and altruism is one of the humanistic qualities thought to underpin professionalism. However, there has been limited exploration of medical students’ perceptions of altruism and if/how it is incorporated in to their professional identity. This study explores medical students’ understanding of altruism and whether they felt it was an important part of being a ‘good’ doctor.

Methodology

Semi-structured qualitative one-on-one interviews with nine students from Leeds University Medical School began by asking participants to provide their own definition of altruism and then explored students’ views of altruism in clinical practice and the importance of altruism to being ‘good’ doctors. Students were encouraged to reflect on their clinical experiences. Interview responses were audio-recorded, transcribed and analysed thematically.

Results

Definitions of altruism varied and were often easily shaken upon questioning. Students conceptualised altruism as a spectrum, ranging from a pure to a more pragmatic form. Generally students did not consider altruism as essential to the role of a doctor and saw it as going “above and beyond” duty. However, almost all participants expressed a preference to work with or be an altruistic doctor. Students also repeatedly referred to the idea of the “right balance of altruism” to avoid self-sacrifice to the point of burnout.

Discussion and Conclusions

The variability in medical students’ definitions of altruism echoes the variability of definitions within literature, which may indicate students’ relatively shallow understanding of the concept. Introducing guided reflection on professionalism within the medical curriculum may help to address this by helping students to develop a deeper understanding of professionalism and how it impacts their practice. Our paper supports a move away from the term altruism towards ‘pro-social behaviour’, which places importance on the benefits of an action without encouraging self-sacrifice. This may continue to provide students with the motivation they associate with altruism, whilst encouraging self-care and work-life balance.

## Introduction

Over the past few decades professionalism has become increasingly recognised as a vital component of medical practice ( Blackall et al., 2007), and its inclusion in seminal publications such as *Tomorrow’s Doctors* ( General Medical Council, 2009) has further ensured its place at the centre of medical education in the UK. Professionalism has been linked to employee morale and productivity, alongside improved communication and teamwork ( Mueller, 2009). Professionalism has also been associated with ensuring patients’ trust in doctors ( Hilton and Slotnick, 2005).

As a result of its increasing importance, medical students are encouraged to develop their professional identities from the very start of their training. Professional identity is the way someone perceives themselves as a doctor ( Wilson, 2013). Forming this identity is a complex process in which students gradually internalise the values and behaviours of the medical profession ( Cavenagh et al., 2000; Holden et al., 2012; Monrouxe, 2010; Smith, 2005) through interaction with the intended and hidden curricula ( Monrouxe, 2010).

One model of professionalism places humanism at the foundation of many professional values. According to Dr Jordan Cohen, professionalism is “a way of *acting*” ( Cohen, 2007, p. 1029) whereas humanism is more a “way of *being*” that “ *animates authentic professionalism*” ( Cohen, 2007, p. 1029). The Arnold P. Gold Foundation identifies the core characteristics of humanism as: respect, altruism, integrity, excellence, compassion and service ( Buck et al., 2015). Of these, altruism was cited by students as the second most important attribute of professionalism ( Sullivan et al., 2014). Some sources go so far as to call altruism a “cornerstone of the Hippocratic oath” ( McGaghie et al., 2002, p. 374).

However, this model is not universally accepted and the importance of including altruism within medical professionalism is debated within literature ( Coulter et al., 2007; Downie, 2002; Glannon and Ross, 2002; Harris, 2017). Despite this, altruistic motives have been reported to rank highly amongst students’ motivation to study medicine ( Crossley and Mubarik, 2002; Gasiorowski et al., 2015; Pagnin et al., 2013). This results in altruism being one of the most debated components within the conception of professional identity - yet it remains the least explored in medical education ( Buck et al., 2015).

There has been limited exploration of medical students’ perceptions of altruism and if/how it is incorporated in to their professional identity. Previous studies have generally tried to quantify students’ altruism levels ( Coulter et al., 2007; Crossley et al., 2002), rather than exploring students’ perceptions of altruism and its importance to their practice. As such, this study aimed to address this gap in the literature, by focusing on exploring Leeds’ medical students’ understandings of altruism and to what extent they felt altruism was part of a doctor’s role and identity. We hoped to gain insight in to what students considered the priorities and values of the medical profession to be, as well as an insight in to their own professional identities and the influences to which they had been exposed.

## Aims

This study’s aims were to:

1. Explore medical students’ understanding of altruism
2. Explore medical students’ experiences of altruism in the clinical setting
3. Explore the extent to which medical students felt altruism was an important aspect of being a doctor

## Methods

### Rationale

This study utilised a qualitative research method in the form of semi-structured interviews. Interviews focused on encouraging students to reflect on their experiences of medical school and enabled an in-depth exploration of participants’ perspectives on altruism. It allowed for a more comprehensive understanding of participants’ world-view than either questionnaires or focus groups. Furthermore, one-to-one interviews were considered to be more appropriate for the topic, as there was a small risk that participants may disclose examples of poor practice, and confidentiality could not have been guaranteed in a group setting.

The semi-structured approach further allowed exploration of ambiguous or insightful answers, and the provision of hypothetical scenarios for participants who struggled to reflect on personal experience.

### Ethical considerations

Ethical approval was granted by the School of Medicine Research Ethics Committee (approval number MREC16-053). A plan was created to address the disclosure of harmful practice. This plan did not need to be actioned.

### Sample and recruitment

The study was advertised to the student body via email and by posts on the lead researchers’ (BS and KM) Facebook accounts. Students of all years could take part, providing they had experienced clinical placement. Participants self-selected by emailing BS or KM. Nine students responded ( Figure 1). All participants were given access to a participant information sheet and consent form prior to the interviews, and on the day of the interview itself. All participants signed consent forms.

**Figure 1:** 

Year	Number of participants
1	0
2	0
3	1
Intercalating	1
4	6
5	1

### Data collection

Interviews lasted between twenty minutes and one hour (depending on the participant’s ability to expand on their thoughts and interest in the research topic) and were undertaken by either BS or KM.

The interviews were guided by a set of pre-determined questions exploring various elements of altruism. Each interview began by asking participants to provide their own definition of altruism. Further questions explored students’ views of altruism in clinical practice, public expectations of altruism in doctors, the importance of altruism to being a ‘good’ doctor, differences in altruism between different healthcare professionals as well as if and how their medical school encouraged altruism in its students.

All interviews were audio-recorded, transcribed verbatim and saved on the password protected university hard drive before being anonymized for analysis. A pilot interview took place, conducted by KM. BS listened to the audio-recording of this interview. Both researchers then discussed the quality of the questions. No changes were made to the initial interview schedule. The pilot interview was not included for analysis.

### Transcription and analysis

Transcripts were analysed thematically. This was a process in which sections of the transcripts containing similar content were grouped and given a descriptive title, known as a ‘theme’. BS and KM analysed all nine transcripts independently and then discussed themes that they had found. There was a high degree of similarity. Differences were noted and explored through discussion, before agreement on the final themes below.

## Results

The results will be presented according to two overarching themes focusing on students’ definitions of altruism as a concept and how this relates to their professional identity as doctors.

### Students’ definitions of altruism

#### Cognitive dissonance

Although participants provided a confident definition of altruism initially, most struggled to elucidate their definition when it was discussed and challenged. Their ideas were also highly malleable. When asked to explore the subtleties of their definition one participant stated:

I don’t think I really understand my own opinion on what the word altruism means, I think that I’ve become quite confused (P3(B))

One participant used the term “cognitive dissonance” to describe their confusion - this is a state in which someone has conflicting beliefs which causes them discomfort and alters their beliefs as necessary ( McLeod, 2014). Participants often revised their definition multiple times as the interview progressed, occasionally contradicting aspects of their initial definition.

#### Altruism as a scale

Participants did not appear to think of altruism as a dichotomous construct in which something was altruistic or not, but referred to altruism as a “spectrum” or “scale”. Where something was positioned on the scale was dependent on the degree to which the benefit to the recipient was off-set by the benefit to the person undertaking the act (agent). At the highest end of the scale was a “pure” or “true form of” altruism, something which benefits the recipient but has no benefit for the agent, or is to the agent’s detriment. Many felt that this theoretical altruism was not possible to achieve. Further down the spectrum lay a “practical definition” of altruism, which most participants felt was more realistic in practice. This form of altruism was defined as benefitting the recipient but also having some *unintended* benefit to the agent, usually in the form of feeling good about themselves for helping another:

[..] there’s like a true form of altruism which isn’t really possible because it’s [..] I don’t see it’s possible to have no gain at all (P3(B))

I don’t think it’s possible to be purely altruistic [..] you can’t really ever do something completely without the knowledge of not having a benefit to yourself (P4(K))

#### Intention vs action

Although participants largely agreed on altruism as driven by selflessness there was disagreement about whether this manifested itself within the intention of the act or the execution of the act. For some, altruism was defined by the purity of the motive and was therefore more of an attitude of daily working:

I think a big part of how I understand altruism is intention. As I say, it’s more than outcome, it’s intention (P1(K))

For others, however, altruism could only be ascribed to actions and not people, thereby implying that it is the act itself which determines how altruistic it is. One participant stated that only others can identify your actions as altruistic. This interpretation removes intention from the equation altogether, as it would be impossible for an outside party to accurately judge the intention of an act. Another differentiated between intention and action by labelling the former as “empathetic” and the latter as “altruistic”:

[..] you can think about doing something, if you’re not gonna do it then you’re not being altruistic [..] you can be empathetic but you won’t be.. you won’t be altruistic (P4(K))

### Altruism and being a ‘good’ doctor

#### Going “above and beyond”

Most participants thought that the day-to-day job of a doctor was not related to altruism and was merely exchanging skills for a service like any other career:

I s’pose the job of the doctor itself [..] that’s exchanging your services for money, which is how the whole of society works (P2(B))

For a doctors’ actions to be altruistic, they had to go “above and beyond” their paid duties. All participants used this term and students frequently referred to the same few examples: time as a commodity, holistic care and carrying out small actions which were not seen as crucial to the care of the patient but would facilitate their treatment journey.

All participants cited instances where doctors sacrificed their time either by staying late after a shift, fitting extra patients into clinics and therefore extending clinic hours, or working through breaks:

Just simple stuff, like, um, you know, staying late after a shift, or something, you know, to go the extra mile for a patient or you know, if you’re a GP and your list’s running late, that you stay an extra ten minutes and take the hit to comfort your patient (P2(B))

Addressing patients’ psychosocial needs as opposed to just treating their immediate medical complaint was another example frequently used to denote altruism:

A GP that I was with, she has a type 1 diabetic [..] I think the way the GP deals with her is incredibly altruistic because she gives her [..] psychological support a lot more in regards to her whole family situation [..] I think that other doctors [..] maybe they would just [..] not actually work to the core of the problem (P5(K))

For most participants the definition of ‘duty’ lay in a distinction of whether an action was absolutely necessary. It was felt that altruism does not reside in emergency situations, but small acts that were non-critical to the patient’s care but would improve their healthcare experience:

[..] it’s not something super acute that the patient is going to die imminently, the patient could wait. But in helping them you are doing that because it makes their experience of medicine that little bit better, they get better that little bit quicker (P1(K))

#### Preferable but not a priority

Participants felt that being altruistic was not fundamental to the professional identity of a doctor. Safety and working within competence were perceived as “absolutely fundamental” and were therefore perceived as much more important. Participants felt that doctors could provide safe care without going “above and beyond”. Altruism was a positive but additional quality.

Importantly, participants’ opinions on the necessity of altruism were also affected by their original definition of altruism. One participant equated altruism with “being caring” and consequently felt there was a “minimum” or “innate amount” needed to do the job. Another considered it a much rarer skill, referring to altruism as “gold-standard selflessness”. This participant’s consideration of altruistic acts as “saint-like” meant that she did not feel altruism was widespread amongst doctors:

I think there are more important things a doctor needs [..] there’s a lack of altruism you can get away with in the profession. I don’t think all doctors have that real spark of altruism (P2(K))

This was also reflected in the way that students spoke about the intended curriculum. When discussing whether any elements encouraged student altruism, some participants appeared to attribute lower levels of value to these experiences compared to, for example, clinical placement.

If I was creating a medical school it [altruism] probably wouldn’t be my priority [..] I don’t think it necessarily breeds good doctors [..] I think those things [lectures by patients] are nice [..] But actually I gain a lot more from going and seeing that patient on the ward [..] it’s not the same exposure I think in terms of really understanding how ill health affects a human (P1(K))

However, all participants agreed that they would prefer to work with/hire/be an altruistic doctor. They referred to the altruistic doctor as one “spoken highly of by patients” (P4(K)), “exceptional” and “excellent”. Across interviews there was a general sense of being drawn to altruistic individuals on a “human level”. As one participant remarked:

I think you don’t have to be altruistic to be a good doctor, I think if you’re more altruistic you’re a “better” person [..] if I had two people whose CVs were identical [..] I would rather hire the altruistic one [..] I think probably just on a human level I would like that human better. I don’t necessarily think they would be better at the job (P1(K))

#### Protective Vs Destructive

Similar to participants’ conception of an altruistic “spectrum” it was felt that doctors need to find the “right” balance of altruism in their practice. Too little altruism would result in disillusionment with medicine, and in this way altruism was seen as a protective factor against frustration and mental exhaustion in a generation where participants felt the financial and social rewards of Medicine had greatly declined. External motivation via contractual obligation was not enough:

[talking about doctors who were less altruistic] it’ll be more difficult for them at this time to thrive [..] if you don’t have this selfless concern for the wellbeing of others, then you’re gonna become increasingly frustrated at a much faster rate about your working environment (P1(B))

The most commonly identified risk of being too altruistic was burnout - emotional, mental and physical exhaustion secondary to occupational stress ( McCray, 2008). An additional risk was that one may get too involved in patients’ lives. This was considered unprofessional and detrimental to doctors’ wellbeing. Students recognised doctors’ responsibility to look after themselves and the risks to patient care if they did not. In these cases *limited* altruism was seen as an important aspect of professionalism in order to provide a “barrier” between the physician and their patient:

If you put too much of yourself in to other people you lose track of yourself [..] you start taking on too much of a burden, it’s important to look after yourself [..] If you don’t look after yourself your mind in itself.. you might be depressed, you might not be sleeping, you might be making mistakes (P4(K))

The idea of a perfect balance within altruism is well supported. Altruism has been linked to improved health, wellbeing, compassion and motivation levels ( Hafler et al., 2011; Post, 2005). However, these benefits can be compromised if individuals become overwhelmed by over-helping ( Burks and Kobus, 2012; Post, 2005). It appears that if altruism *is* to be part of professional identity, there is a “right” amount that doctors must have.

## Discussion

The authors found that students’ definitions of altruism were as variable as definitions of altruism in the current literature. Definitions of altruism as determined by action echoes iterations of altruism as a behaviour ( Coulter et al., 2007; Glannon and Ross, 2002; Mueller, 2009). However, many students conceptualised altruism as an intention. Alongside a wide variation in definitions it is important to note the difficulty with which participants’ elucidated their definitions of altruism. The range of interpretations our research uncovered supports the notion that concepts such as altruism and professionalism are abstract and nebulous in nature ( Mueller, 2009), and therefore difficult to define exactly. Although it is possible that participants struggled to maintain consistency because of this nebulous nature ( Burks and Kobus, 2012), it may also have been the result of their junior status. Developing a professional identity involves integrating ones’ professional and personal identities. This is a process of continual development and can take many years ( Hilton et al., 2005; Monrouxe, 2010). The participants were early-on in this process. This may also explain the finding that most participants had seemingly never considered the concept of altruism very deeply and were challenged by the interview to critically appraise and develop their understanding.

A guide released by the Association for Medical Education in Europe (AMEE) for integrating professionalism into the curriculum instructed that students require “discussion and guidance” ( O’Sullivan et al., 2012, p. e69) when reflecting on clinical experiences. This would help to address certain negative experiences within the ‘hidden curriculum’, which have been associated with loss of idealism and altered ethical integrity ( Lempp and Seale, 2004), by exposing their influence on students’ perspectives and allowing issues to be addressed in the formal curriculum ( Chalmers et al., 2011). Furthermore, it would allow students’ opinions to be either incorporated into an ever-evolving conceptualisation of professional values or, if inappropriate, to be “brought in line” ( O’Sullivan et al., 2012, p. e68) with the values of their institution.

One-to-one discussions have been shown to be more effective in aiding reflection on professionalism than writing tasks, allowing for in-depth exploration of the topic ( Baernstein and Fryer-Edwards, 2003). Vivekananda-Schmidt *et al* (2011) reported that students found informal discussions with peers and tutors beneficial, whereas they were more conscious of their reflection in written tasks secondary to tension between the private nature of their thoughts and an inability to know who could read these personal feelings. A 2003 study at the University of Sydney introduced a personal and professional development strand to its first year medical students which involved completing a portfolio and confidential interview with a member of staff ( Gordon, 2003). Its design as a creative but formative assessment allowed for students’ creativity whilst ensuring student engagement, and the overwhelming majority found the exercise engaged them in useful reflection on their course ( Gordon, 2003). The authors propose a similar intervention be trialed in the UK to allow students to engage more fully with the concepts of professionalism and contributive elements such as altruism. Such reflective practice has been shown to benefit the arena of healthcare more generally, leading to improved well-being in doctors, improved patient care and patient centeredness and improved teamwork ( Lutz et al., 2013).

Given the general uncertainty surrounding altruism, and the risk of burnout due to its association with self-sacrifice, this could be considered an unstable foundation from which to build students’ understanding of professionalism. This paper supports literature promoting alternative terms in medical education such as ‘pro-social behaviour’. ‘Pro-social behaviour’ is a categorisation of behaviours that focus on the positive benefits for the recipient ( Wispé, 1972) without demanding either self-sacrifice from, or a lack of gain for the helper ( Burks and Kobus, 2012). Furthermore, pro-sociality’s focus on the welfare of others continues to provide students with the motivation they felt was necessary to overcome struggles within clinical practice. However, unlike altruism, no interpretations of pro-sociality perpetuate the idea of detriment to the agent. Promoting pro-social behaviour over altruism may therefore minimise the risk of burnout and support the increasing focus on self-care and work-life balance ( Bishop and Rees, 2007; Fresa-Dillon et al., 2004). Self-care is not only important for the wellbeing of doctors but that of patients as well, as better mental health in doctors has been proven to have a positive impact on patient care ( Dyrbye et al., 2010; Gordon, 2003; Sanchez-Reilly et al., 2013).

## Reflexivity and Limitations

It is important to note that with all qualitative studies, some of participants’ views may have been the result of social desirability bias (Psychology Concepts, 2011). Participants may have been unwilling to admit to the researchers (and potentially themselves) some authentic views about their perceptions of professional identity, humanism and altruism. Participants may have provided answers that would make themselves sound favourable, or that would be in-keeping with what they thought the interviewers wanted to hear. BS and KM were medical students at the University of Leeds at the time of the interviews, so had a shared experience of the curriculum with the participants. As such, participants may have felt more comfortable talking about their experiences and ideas, or may have felt they could not express negative points of view in front of someone they knew. Alternatively, they may also have conformed to more ‘cynical’ points of view, or provided answers more in-keeping with the culture at Leeds Medical School. To try and reduce the incidence of the latter BS and KM assured participants that they could express negative thoughts if they felt it was necessary. They also reiterated that all data would be anonymized. With regards to analysis, both authors had limited pre-conceived ideas around the notion of altruism, and had not formulated their own definition. This allowed them to work through students’ definitions from various stand-points, rather than one defined approach.

## Conclusions

Despite altruism being cited as a strong motivation for studying medicine, student participants in this study generally displayed a shallow understanding of altruism which was easily shaken by interviewers’ questions. This may reflect the complexity of the concept or students’ junior status. Medical schools should encourage individual reflection to allow students to reflect on altruism as well as potentially negative influences of the hidden curriculum. Students also reinforced the relationship between altruism and a risk of burnout. As such, this paper supports literature emphasizing a move away from the term ‘altruism’ to ‘pro-sociality’, as the focus on the welfare of the patient *and* the doctor, which hopefully allows medical students to remain motivated whilst avoiding burnout and emotional over-involvement.

## Notes On Contributors

The first two authors of this manuscript are jointly responsible for the work and should therefore be considered joint first authors of the paper.

Kaat Marynissen is a final year medical student at the University of Leeds with an interest in the medical humanities and their application to medical education. She holds a BSc in Medical Humanities from the University of Glasgow.

Bethan Spurrier is a final year medical student at the University of Leeds with an interest in medical education, particularly the development of assessment tools. She holds a BSc in Medical Education from the University of Leeds.
