# Supplementary material for: Becoming the 'good doctor': medical students’ views on altruism and professional identity
Source: MedEdPublish (2016). 2018 Mar 6;7:52. [Version 1] doi: 10.15694/mep.2018.0000052.1 (PMC10711959; doi:10.15694/mep.2018.0000052.1)
Supplement: Peer review history

# Reviewer report: Reviewer response for version 1

**DOI:** 10.21956/mep.19530.r28910

**Published:** 2018-03-08

**Authors:** Tan Nguyen

**Affiliations:** 1 Deakin University

This review has been migrated. The reviewer awarded 4 stars out of 5 I thoroughly enjoyed this paper. Often there is a cognitive dissonance between the student learner and clinical educator's understanding of altruism. The research could be strengthened by outlining the theory underpinning the qualitative research, and perhaps providing an indication by the researchers if saturation of the themes were reached. Further discussion on the limitations of the research would add value as well. eg. generalisability, comparisons to other research in the area, potential impact of power relationships, implications for practice etc.

Reviewer Expertise:

NA

No decision status is available

# Reviewer report: Reviewer response for version 1

**DOI:** 10.21956/mep.19530.r28909

**Published:** 2018-03-06

**Authors:** Trevor Gibbs

**Affiliations:** 1 AMEE

This review has been migrated. The reviewer awarded 4 stars out of 5 A very interesting paper about a rather difficult situation.Much is spoken of altruism, the word frequently appearing in schools' mission statement and sometimes in specific learning outcomes.I have even heard it spoken of in pre-medical school interviews. I agree with the authors that it probably is the poor cousin of professionalism and appears much more as words rather than action. - the challenge being placing it in a rightful place in a curriculum. I was warmed when I saw that this paper was written by two medical students who had clearly put a lot of work into it- great references - and I hope they continue with their work in this area, maybe expand the numbers researched. A very good paper to recommend.

Reviewer Expertise:

NA

No decision status is available
